# Primary abdominal gas gangrene: a report of two autopsy cases

**DOI:** 10.4322/acr.2021.329

**Published:** 2021-09-23

**Authors:** George S. Stoyanov, Deyan L. Dzhenkov, Lilyana Petkova

**Affiliations:** 1 Medical University - Varna “Prof. Dr. Paraskev Stoyanov”, Faculty of Medicine, Department of General and Clinical Pathology, Forensic Medicine and Deontology, Varna, Bulgaria

**Keywords:** Gas Gangrene, Abdomen, Clostridium, Liver, Histology, Pathology, Autopsy

## Abstract

Primary hepatic gas gangrene is a form of primary abdominal gas gangrene. The condition is caused by *Clostridium perfringens*, other clostridia, and non-clostridia bacterial species producing gas. Unlike classical gas gangrene or myonecrosis, the disease develops without a wound or a port of entry. Instead, gas-producing bacteria in the gastrointestinal tract colonize an underlying pathological process with foci of necrosis, producing excessive gas and spreading hematogenously to other organs. Herein we present two autopsy cases of primary hepatic gas gangrene diagnosed on autopsy, with the gross and histological changes that can be considered specific for this rare condition. Both patients had severe underlying liver disease-prone for this entity development. The gross changes in the cases are postmortem subcutaneous emphysema, skin bullae with pooled blood, pneumothorax, pneumoabdomen, abundant gas in the circulatory system, porous structure of the internal organs (tissue gas bubbles), and advanced tissue lysis, not corresponding to the post mortem time. Histology showed optically empty areas of varying size in the internal organs, which weave the structure of the organs and rod-shaped bacteria with scarcity or complete absence of inflammatory reaction.

## INTRODUCTION

Gas gangrene is an infection caused predominantly by *Clostridium perfringens* and is most commonly associated with soft tissue infections of the extremities after trauma.^1^^-^ 4 This entity is also known as bacterial myonecrosis.^1^ Non-traumatic gas gangrene is rare, and most commonly, the site of the primary infection is in the abdominal cavity, involving predominantly the liver, pancreas, or gallbladder. It is frequently associated with malignancy, immunosuppression, or severe organic degenerative changes.^3^^,^ 5 ^-^ 8 Herein, we report two autopsy cases of primary hepatic gas gangrene.

## CASE 1

The patient was a 62-year-old caucasian male complaining of dizziness, headache, and nausea, without vomiting. Previous medical history included type 2 diabetes mellitus for 23 years, hypertension, ischemic heart disease, chronic renal failure, and benign prostate hyperplasia. Laboratory tests are summarized in Table 1. The physical examination showed the heart rate of 85 beats per minute (bpm), arterial pressure (BP) of 160/80mmHm. Examination of the respiratory system was unremarkable, and lower limbs pitting edema was absent; however, the abdomen was distended, painless without any palpable mass.

**Table 1:** Laboratory findings of both patients

Test	Case 1	Case 2	reference value
hemoglobin	11.3	18.4	12.0-16.0 g/dL
leucocytes	4.12	1.43	4-10 10*9/l
platelets	128	68	140-440 10*9/l
urea	30.24	14.56	8.9-23 mg/dL
creatinine	1.97	2.15	0.5-1.1 mg/dL
glucose	280	280	< 100 mg/dL
AST	23.6	29.2	0-34 U/l
ALT	24.3	30.2	10-49 U/l
C-RP	63.88	165.13	0-5 mg/ml

AST: aspartate aminotransferase; ALT: alanine aminotransferase; C-RP: C-reactive protein.

Due to the neurological symptoms, the patient was referred to a neurological evaluation which reported severe dysphonia, dysarthria, dysmetria, decreased spinal reflexes, and a positive Babinski reflex. The patient was referred for brain computer tomography (CT). However, the patients' condition deteriorated. The accompanying neurologist reported a Glasgow coma scale of 3 points, and a perceived shock condition was suspected. Cardiopulmonary resuscitation (CPR) was initiated without success. The patient was pronounced dead thirty minutes after the initial presentation.

## CASE 2

A 62-year-old caucasian female sought medical care in a severely deteriorated clinical condition with complaints of general malaise, dyspnea, abdominal pain, and vomiting over the last three days prior. Previous medical history included breast cancer with liver metastases. Laboratory tests are summarized in Table 1. Her clinical examination revealed a heart rate of 90 bpm, BP 60/30 mmHm, facial cyanosis, severe jugular stasis, no peristalsis, and a palpable liver 3cm below the costal border. A shock condition was suspected. Intensive care measures were Initiated but proved ineffective, heart rate on the fifth minute after the presentation was 0 BPM, and BP was nondetectable. CRP protocols were initiated but were ineffective as well. The patient was pronounced dead fifteen minutes after the presentation.

### Laboratory findings

Both patients showed elevated levels of serum glucose, creatinine, and c-reactive protein (CRP). A more detailed list of laboratory parameters is shown in Table 1.

## AUTOPSY PRESENTATION

**Figure 1:**
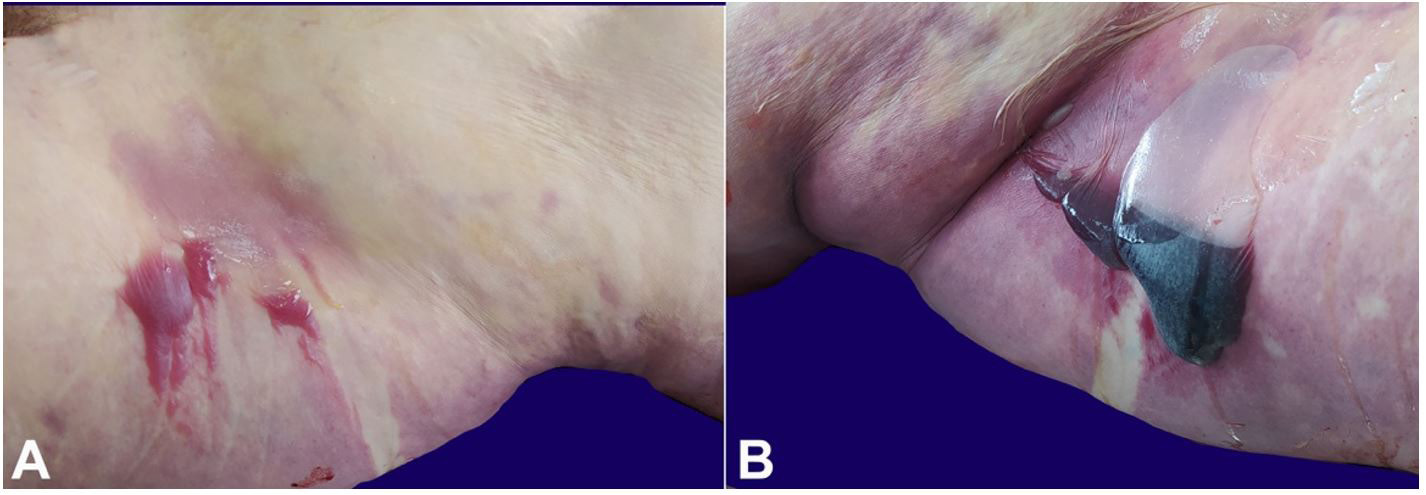
Skin bullae; **A** – bullae in the first patient; **B** – bulla with pooled blood in the second patient.

**Figure 2:**
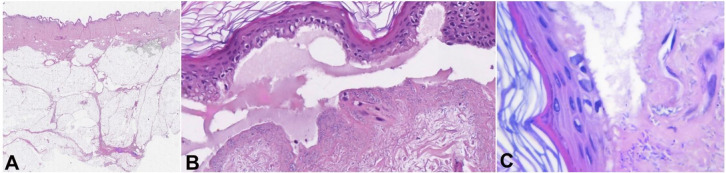
**A** – histopathology from a bulla in the second case with epidermal delamination, without inflammatory changes (H&E, 2x); **B** – high power magnification from (**A**) showing subepidermal detachment with the accumulation of protein-rich fluid in the bullous cavity and dermal proliferation of rod-shaped bacteria without inflammatory reaction (H&E, 400x); **C** – rod-shaped bacteria in the dermis (H&E,1000x).

Both autopsies were performed within eight hours after the death. Both patients had a body mass index (BMI) of more than 35. Subcutaneous crepitations corresponding to subcutaneous emphysema were noted in both patients in all topographical areas. Abdomen and lower limbs showed skin bullae, some of which with pooled blood (Figures 1 and 2).

Rigor mortis had been set in both patients across all muscle groups except the palmar muscles. On section, free gas, under pressure, was present in the subcutaneous tissue and the thoracic and abdominal cavities. Therefore, a probe for gas and air embolism was attempted. Still, upon filling the pericardial pouch with water and severing the cardiac chambers, large gas bubbles were released from all four cardiac chambers. Both patients had gross changes associated with hypertension - cardiomegaly (cardiac weight 520 g (mean reference range [mRR] 302 g);, left ventricular thickness 19mm (MRR; 11,5 mm) and right ventricular thickness 7mm (mRR; 4 mm) in the first patient, and in the second case the cardiac weight was 440g (mRR; 302 g), left ventricular thickness 17mm (mRR; 11,5) and right ventricular thickness 5mm (mRR; 4 mm), and hypertensive nephrosclerosis.

All internal organs were greyish, soft, and cross-sections revealed a porous surface most pronounced in the liver and spleen. In addition, both patients had enlarged steatotic livers, with multiple metastases in the second case.

All organs seemed to have undergone severe autolysis, not corresponding to the time elapsed after death and the presence of *rigor mortis*.

In Case 1, other gross changes worthy of note were a small polypoid tumor of the cecum, and in case 2, multiple liver metastases were present.

Specimens of all internal organs were obtained and preserved in 10% buffered formaldehyde for histological evaluation.

### Histopathology

**Figure 3:**
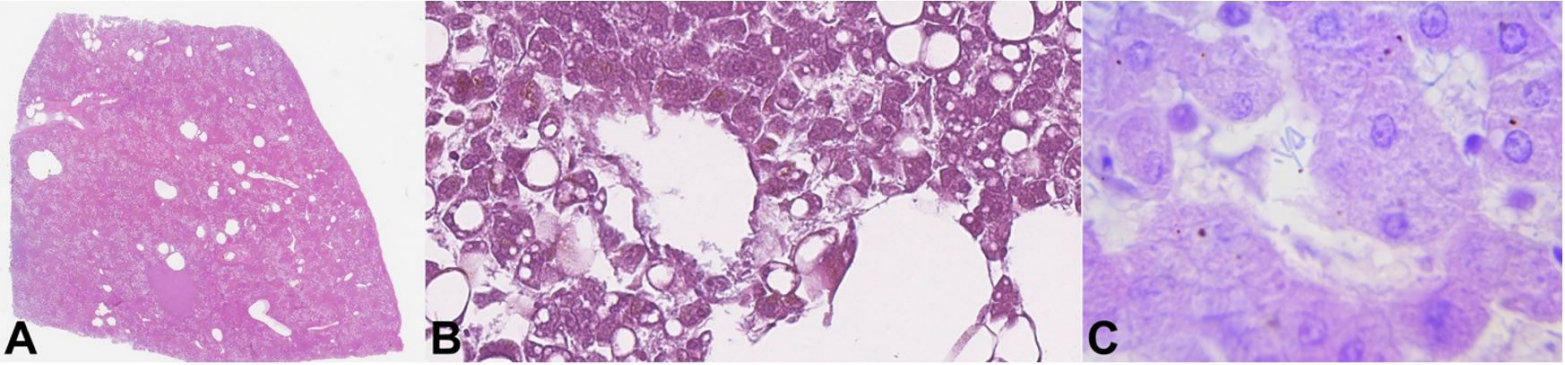
Histopathology of the liver from the first case. **A** – multiple optically empty areas, with varying size (H&E, 2x); **B** – rod-shaped bacteria in adjacent liver parenchyma without inflammatory reaction (H&E, 400x); **C** – rod-shaped bacteria in the liver parenchyma (H&E,1000x).

**Figure 4:**
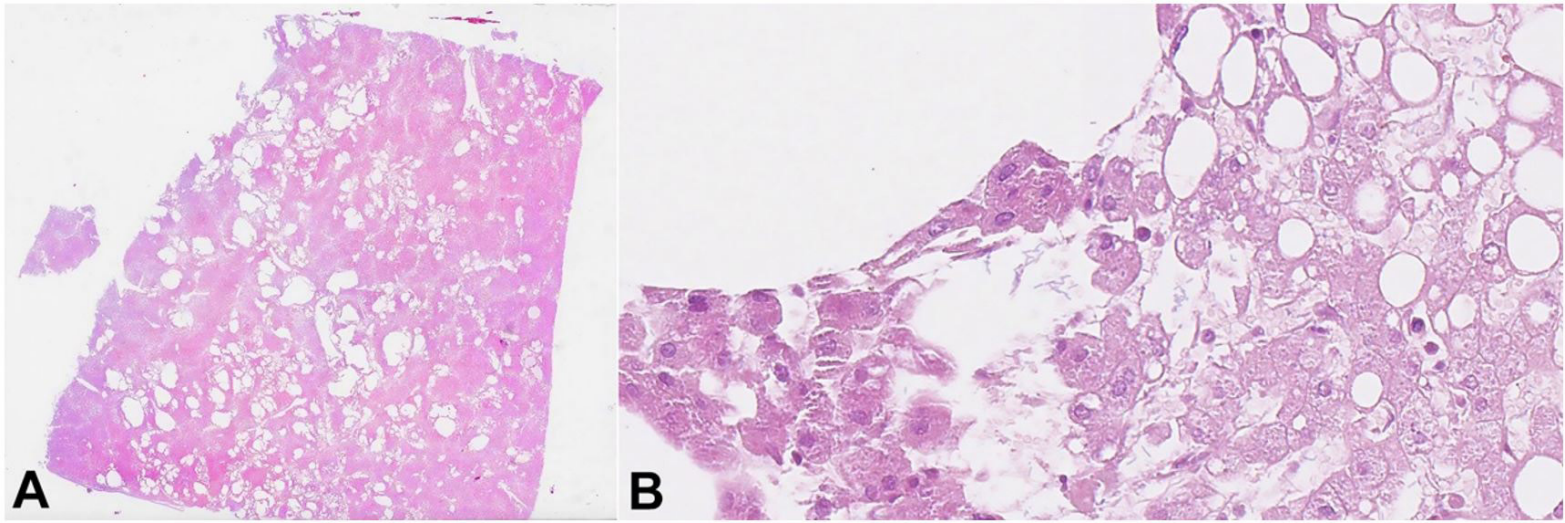
Histopathology of the liver from the second case. **A** – multiple large optically empty areas (H&E, 2x); **B** – rod-shaped bacteria in adjacent liver parenchyma without inflammatory reaction (H&E, 400x).

Histopathology from both cases revealed a porous composition of the organs, with varying pore size, from small to medium and large optically empty areas dissecting the architecture of the organs and artificially weaving its structure. These correspond grossly to gas-filled bubbles. In most of these optically empty areas, free rod-shaped bacteria and rod-shaped bacterial colonies could be seen, as well as rod-shaped bacteria in the adjacent tissue, with a paucity or complete lack of inflammatory cells. In both patients, the most severely affected organ was the steatotic liver (Figures 3, 4).

**Figure 5:**
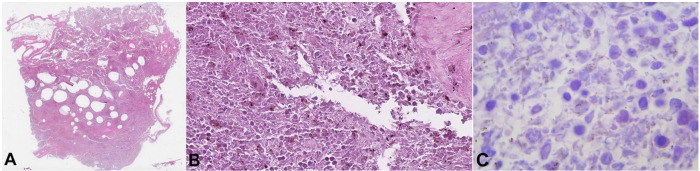
Histopathology of the spleen from the first case. **A** – multiple large optically empty areas (H&E, 2x); **B** – rod-shaped bacteria in adjacent parenchyma and bacterial colonies without inflammatory reaction (H&E 400x); **C** – rod-shaped bacteria in spleen parenchyma (H&E,1000x).

**Figure 6:**
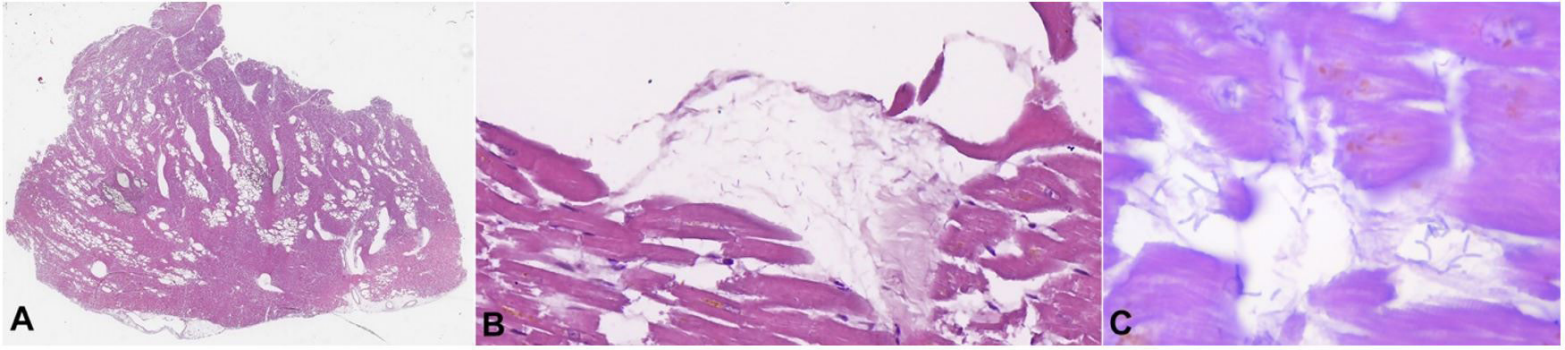
Histopathology of the myocardium from the second case. **A** – multiple optically empty areas, with varying size (H&E, 2x); **B** – rod-shaped bacteria in adjacent parenchyma without inflammatory reaction (H&E 400x); **C** – rod-shaped bacteria in the myocardium, H&E 1000x).

In both cases, similar changes were observed in multiple organs, namely the spleen, pancreas, gastrointestinal tract mucosa, kidneys, and myocardium (Figures 5, 6).

In both cases, additional unterated to the gas gangrene findings were cardiomyocyte hypertrophy, with scant interstitial myocardiosclerosis, arteriolohyalinosis, and sclerosis in the kidneys (with glomerulosclerosis) and brain, corresponding to hypertension, as well as fibrosis and lipomatosis of the pancreas.

### Correlation between the morphological and clinical data

In both cases, the treating physicians were consulted for the presence of the bullae and subcutaneous emphysema. In both cases, they confirmed these changes were not present antemortem.

The hepatic gas gangrene diagnosis was established based on the fulminant clinical course, gas-filled bubbles throughout the organs, and advanced lytic changes, while *rigor mortis* was still set. Underlying conditions for development were considered severe steatosis in the first patient and multiple hepatic metastases from the mammary carcinoma in the second patient.

## DISCUSSION

Primary hepatic gas gangrene and primary abdominal gas gangrene are rapidly fatal and underreported conditions, difficult to recognize and treat clinically.^3^^,^ 8 In contrast to wound gas gangrene, which is easily suspected in the case of a superficial wound, no specific morphological changes may be found to aid the diagnosis.^2^^,^ 3 As seen in our patients, the presenting symptoms were unspecific and fulminantly progressed to shock, which did not allow for proper examination and the identification of the infection.

The postmortem interpretation in such cases should be cautious and detailed. The physicians treating the patients' should be consulted for the postmortem development or progression of subcutaneous emphysema, bullae, and the presence of localized organ symptoms, or if present imaging investigations such as computer tomography (CT) or abdominal ultrasound.^3^^,^ 6 ^,^ 8 ^-^ 10 The only somewhat specific gross change that can be considered in such cases is the presence of advanced parenchymal organ autolysis, while rigor mortis is still present.^5^ Therefore the interpretation of these changes is difficult in autopsies which take place more than 48 hours after the patient has died.^5^^,^ 11 By that postmortem period, similar changes should be interpreted as putrefaction, producing identical organ changes.^11^ However, it usually requires more than a week to reach the severity seen in our cases and others described in the literature.^11^ The development of skin bullae with pooled blood in a short postmortem period results from the gas from the subcutaneous emphysema dissecting the dermo-epidermal border. However, again clinical correlation should be very detailed.^11^ The only histologically distinctive features of the disease are large optically empty areas spread throughout the internal organs, rod-shaped bacteria, scarcity of inflammatory reaction due to the fulminant outcome, and postmortem progression.^1^^,^ 4 ^,^ 11

The site of primary development in the liver and adjacent structures is likely due to clostridia and other gas-producing bacterial species in the gastrointestinal tract (GIT).^3^^,^ 5 ^,^ 8 ^,^ 12 ^-^ 15 However, it should be noted that primary gastrointestinal tract infection with Clostridium perfringens causes symptoms of food poisoning and not gas gangrene.^12^ The condition needs a severe background in the GIT, predominantly with foci of necrosis, to develop as gas gangrene in people already carriers of the bacteria.^5^^,^ 8 ^,^ 12 Cases of iatrogenic development have also been reported after surgical interventions, including decades after a liver transplant and after intestinal ruptures following endoscopy.^4^

Postmortem microbiological isolation of clostridia and other gas-producing bacteria can be of aid.^4^^,^ 8 ^,^ 10 ^,^ 16 Still, only in the context of morphology and postmortem time, as even without active infection, gas production by clostridia is a natural process.^4^^,^ 10 ^,^ 11 ^,^ 15 ^,^ 17

Clinically although difficult to suspect and diagnose, a combination of abdominal CT or ultrasound do identify gas collections in parenchymal organs as well as microbiological identification of clostridia, and antimicrobial treatment has been reported to still be with high mortality due to the progressive nature of the condition.^3^^,^ 6 ^,^ 8 ^-^ 10 ^,^ 14 ^-^ 16

## CONCLUSION

Primary hepatic gas gangrene, as a form of primary abdominal gas gangrene, is a rare condition, difficult to diagnose clinically and with high mortality. However, the two autopsy cases presented herein underline the gross and morphological changes of the internal organs, which can be considered specific in the first 48 hours postmortem.
